# Evaluating the Impact of Attention Mechanisms on a Fine-Tuned Neural Network for Magnetic Resonance Imaging Tumor Classification: A Comparative Analysis

**DOI:** 10.7759/cureus.80872

**Published:** 2025-03-20

**Authors:** Kian A Huang, Neelesh Prakash

**Affiliations:** 1 Radiology, University of South Florida (USF) Health Morsani College of Medicine, Tampa, USA

**Keywords:** attention mechanism, computer vision, deep learning artificial intelligence, magnetic resonance imaging, tumor imaging

## Abstract

Background

Magnetic resonance imaging (MRI) is essential for brain tumor diagnosis. Deep learning models, such as Residual Network 50 Version 2 (ResNet50V2), have demonstrated strong performance in tumor classification. However, integrating attention mechanisms may further enhance diagnostic accuracy. This study evaluates the impact of different attention mechanisms on a ResNet50V2-based MRI tumor classification model for distinguishing between meningioma, glioma, pituitary tumors, and cases with no tumor.

Methods

A ResNet50V2-based model was trained on 3,096 annotated MRI scans from a publicly available dataset on Kaggle. Five model configurations were evaluated: baseline ResNet50V2, Squeeze-and-Excitation (SE), Convolutional Block Attention Module (CBAM), Self-Attention (SA), and Attention Gated Network (AGNet). Performance was assessed using accuracy, area under the receiver operating characteristic curve (AUC), precision, and recall. Two-proportion Z-tests were conducted to compare classification accuracies among models.

Results

The SE-enhanced model achieved the highest classification performance, with an accuracy of 98.4% and an AUC of 1.00, outperforming the base ResNet50V2 (92.6%) and other attention-based frameworks (CBAM: 93.5%, SA: 91.6%, AGNet: 94.2%). Compared to the baseline model, the SE model also demonstrated improved meningioma and pituitary tumor classification (Z = 2.485, p = 0.013 and Z = 2.423, p = 0.015, respectively). Additionally, the SE model demonstrated superior precision and recall across all tumor classes.

Conclusion

Incorporating attention mechanisms significantly improves MRI-based tumor classification, with SE proving to be the most effective. These findings suggest that SE-enhanced models may improve diagnostic accuracy in both research and clinical applications. Future research should explore hybrid attention mechanisms, such as transformer-based models, and their broader applications in medical imaging.

## Introduction

Brain and central nervous system (CNS) tumors are a rare group of heterogeneous cancers that are often symptomatic and life-threatening with poor prognosis [1]. The prevalence of brain and CNS cancer is an estimated 182,520 people in the United States, with an estimated 25,400 new cases of CNS cancer in 2024, accounting for 3.1% of all cancer deaths [2]. The identification and classification of brain tumors are critical tasks in neuro-oncology with earlier diagnoses leading to better outcomes in patient prognosis and survivability [3]. Magnetic resonance imaging (MRI) is a standard imaging modality used for diagnosing brain tumors; however, the manual interpretation of MRI scans can be time-consuming and prone to variability. Recent advancements in artificial intelligence (AI), particularly deep learning, have shown promise in automating image analysis tasks through the use of convolutional neural networks (CNNs) [4]. Transfer learning is a concept of deep learning with CNNs that involves leveraging the skills and pattern recognition of models pre-trained on large general image datasets (e.g., ImageNet) for similar, yet specific tasks [4]. Pre-trained CNN architectures have demonstrated capable medical image recognition, especially on MRI [4,5]. In traditional CNNs, increasing the number of layers can hinder the effective propagation of information from the input to the output. The Residual Network 50 Version 2 (ResNet50V2) architecture addresses this challenge by incorporating skip connections, which enable the direct transfer of information across multiple layers [5]. These connections facilitate more efficient learning and mitigate the vanishing gradient problem, where the training signal diminishes as it propagates through very deep networks. Accordingly, this study will take advantage of the ResNet50v2 architecture.

Another limitation of CNNs is that all feature outputs or feature channels are evaluated equally, lacking a mechanism to prioritize the most informative ones. Additionally, they primarily focus on spatial information without explicitly leveraging global context for feature recalibration [6]. Attention mechanisms in deep learning are designed to enhance CNN performance by dynamically focusing on the most relevant features and enabling models to capture complex dependencies while minimizing the influence of less important information [6]. By selectively prioritizing spatial, channel-wise, or combined feature representations, attention mechanisms improve efficiency, adaptability, and generalization across various tasks such as image classification [6].

In this paper, we explore four different attention strategies including Squeeze-and-Excitation (SE), Convolutional Block Attention Module (CBAM), Self-Attention (SA), and Attention Gated Network (AGNet). Through a squeeze operation, SE blocks aggregate spatial information to create a global channel descriptor, allowing the network to utilize global dependencies [7]. This is followed by an excitation operation, where a self-gating mechanism adaptively reweights feature maps, selectively emphasizing informative channels while suppressing less useful ones [7]. Unlike standard CNNs, which apply fixed feature weightings, SE blocks dynamically adjust feature importance based on the input, enhancing the network's representational power. By integrating SE blocks, CNNs achieve improved performance in visual recognition tasks by better capturing channel relationships and refining feature representations. CBAM integrates both channel attention (which features are important) and spatial attention (which locations are important), allowing models to not only recognize "what" is important but also "where" it is located [6]. SA was originally proposed for natural language processing and later adapted for computer vision in order to capture long-range dependencies between different spatial locations in an image [8]. This mechanism models relationships between distant but related features such as buildings on opposite sides of an image, which allows the network to recognize their correlation and adjust its focus accordingly. In this approach, each pixel's feature representation is computed as a weighted combination of all other pixel features, enabling a more comprehensive understanding of spatial dependencies across an image [8]. AGNet utilizes multi-scale learning, analyzing both fine-grained details and broader contextual information simultaneously, and cross-spatial attention mechanisms, focusing on the most important features in an image by comparing and weighing information across different regions [9]. This study aims to contribute to the CNN-based medical imaging literature by directly evaluating the impact of various attention mechanisms on a ResNet50V2-based MRI tumor classification accuracy. By integrating SE, CBAM, SA, and AGNet, the study aims to identify the most effective approach for enhancing diagnostic performance.

## Materials and methods

The public dataset was obtained from Kaggle (Appendices) and consisted of over 3,000 labeled T1 and T2 MRI images including coronal, sagittal, and axial views representing four classes: meningioma (n = 913), glioma (n = 901), pituitary tumors (n = 844), and no tumor (n = 438). Images were preprocessed by resizing from 256 × 256 to 224 × 224 pixels while preserving aspect ratios, normalizing pixel values, and using horizontal flipping as a method of data augmentation. The dataset was shuffled and split into training (80%), validation (10%), and test (10%) subsets. 

The baseline model utilized ResNet50V2, pre-trained on ImageNet, as the feature extraction backbone. To leverage the generalization capabilities of the pre-trained model, initial layers were frozen, preserving their learned weights to extract fundamental visual patterns. Fine-tuning was enabled by unfreezing deeper model layers, allowing adaptation to the domain-specific characteristics of the MRI tumor dataset. To enhance the model's ability to focus on salient regions and features, various attention mechanisms, including SE, CBAM, SA, and AGNet, were individually incorporated, resulting in four distinct versions of the ResNet50V2 model. These mechanisms aimed to improve feature representation by emphasizing relevant spatial and channel-wise information. After applying the attention block, custom layers and functions including dense, dropout, and global average pooling were added to refine extracted features and facilitate the final tumor classification. The model was trained (epochs = 100, batch size = 16) using the Adam optimizer with an initial learning rate of 0.0001 and categorical cross-entropy for the loss function. Additionally, callbacks such as early stopping and learning rate reduction were implemented to optimize training efficiency and prevent overfitting. To evaluate the performance of the model, multiple metrics were employed to provide a comprehensive assessment of its classification ability. Testing accuracy was used as a primary metric, representing the proportion of correctly classified samples out of the total test set. The area under the receiver operating characteristic curve (AUC) was utilized to measure the model's ability to distinguish between different classes, with an AUC value of 1.0 indicating perfect discriminative performance. The AUC was macro-averaged. Precision was assessed to determine the proportion of correctly predicted positive cases out of all predicted true and false positives. Recall, or sensitivity, was used to measure the proportion of actual positive cases correctly identified from both true positives and false negatives. Finally, the F1 score, the harmonic mean of precision and recall, was included as a balanced metric to account for both false positives and false negatives, making it especially useful in cases of class imbalance. Furthermore, two-proportion tests were conducted to statistically compare the testing accuracies of different attention-enhanced models, providing insights into the impact of each attention mechanism on classification efficacy.

## Results

As shown in Table 1, the SE-enhanced ResNet50V2 model demonstrated the highest overall performance across all configurations, achieving a test accuracy of 98.4% and the highest AUC of 0.999. This model significantly outperformed the baseline ResNet50V2, which had a test accuracy of 92.6% and an AUC of 0.987 (Table 1). Among the attention-enhanced models, AGNet ranked second in overall performance, achieving an accuracy of 94.2% and an AUC of 0.992, followed closely by CBAM with an accuracy of 93.6% and an AUC of 0.993 (Table 1). The SA model had the lowest accuracy among attention-enhanced models at 91.6% with an AUC of 0.988, performing slightly below the baseline in accuracy but maintaining a competitive AUC. In tumor-specific classification, SE achieved the highest accuracy across glioma (98%), meningioma (98%), no tumor (97%), and pituitary tumors (100%), as seen in Table 1. The baseline ResNet50V2 model performed notably lower, with 92% accuracy for glioma, 89% for meningioma, 99% for no tumor, and 93% for pituitary tumors. CBAM and AGNet performed similarly, achieving 95% accuracy for pituitary tumors, whereas SA had the lowest accuracy among the attention-based models for meningioma (87%) and no tumor classification (94%). Notably, the baseline model outperformed SA in meningioma and no tumor classification. Grouping models based on overall performance, SE, AGNet, and CBAM outperformed the baseline ResNet50V2 in accuracy, with improvements ranging from 0.9% to 5.8% (Table 1). Conversely, SA underperformed relative to the baseline, with a 1% decrease in accuracy despite maintaining a competitive AUC. SE was the only model to achieve an AUC of 0.999, while all other attention-enhanced models maintained AUC values above 0.988, demonstrating strong classification capability.

**Table 1:** Test Accuracy and Results by Attention Model Type The table portrays the overall AUC score, model overall testing accuracy, and individual classification accuracy by tumor type. AUC: area under the receiver operating characteristic curve

Models	Total Questions Correct (n = 311)	Test Accuracy	Area Under the Receiver Operating Characteristic Curve	Glioma Accuracy	Total Glioma Questions	Meningioma Accuracy	Total Meningioma Questions	No Tumor Accuracy	Total No Tumor Questions	Pituitary Accuracy	Total Pituitary Questions
Squeeze-and-Excitation	306	0.984	0.999	0.98	97	0.98	93	0.97	40	1	81
Convolutional Block Attention Module	291	0.936	0.993	0.92	93	0.91	87	0.97	53	0.95	78
Attention Gated Network	293	0.942	0.992	0.93	91	0.93	92	0.97	43	0.95	85
Self-Attention	285	0.916	0.988	0.92	102	0.87	84	0.94	44	0.95	81
Baseline Model	288	0.926	0.987	0.92	85	0.89	91	0.99	58	0.93	77

As highlighted in Table 2, a statistical analysis was conducted using a two-proportion Z-test among each pair of models comparing differences in overall testing accuracy and individual tumor classification accuracy (glioma, meningioma, pituitary, and no tumor). P-values of less than 0.05 were considered statistically significant. SPSS version 30 (IBM Corp., Armonk, NY) was used for analysis. The analysis confirmed that the SE model's performance was significantly higher than alternative models across multiple tumor classifications. Statistically significant differences were observed in overall accuracy between the SE and CBAM models (Z = 3.055, p = 0.002) and between the SE and AGNet models (Z = 2.775, p = 0.006). While SE also outperformed SA and the baseline ResNet50V2, the differences were not statistically significant. As shown in Table 2, for tumor-specific classification, significant improvements were noted for meningioma classification accuracy, where the SE model outperformed CBAM (Z = 2.079, p = 0.038), SA (Z = 2.823, p = 0.005), and the baseline ResNet50V2 (Z = 2.485, p = 0.013). Similarly, for pituitary tumor classification, the SE model showed statistically significant gains over CBAM (Z = 2.038, p = 0.042), AGNet (Z = 2.039, p = 0.041), SA (Z = 2.038, p = 0.042), and the baseline ResNet50V2 (Z = 2.423, p = 0.015). Although SE demonstrated higher test statistics for glioma classification, the differences did not reach statistical significance compared to other models (p > 0.05). The classification of "no tumor" cases did not yield any statistically significant differences across models, as all p-values remained above the threshold (p > 0.05). Comparisons between other architectures, such as CBAM versus SA (Z = 0.953, p = 0.341) and AGNet versus SA (Z = 0.501, p = 0.616), did not demonstrate statistical significance in overall accuracy, suggesting relatively similar performances between these attention mechanisms. However, the CBAM model showed notable but non-significant underperformance relative to SE, as indicated by a large negative test statistic (Z = -3.305, p = 0.0019) when compared to the baseline ResNet50V2.

**Table 2:** Two-Proportion Z-Test Analyses Two-proportion Z-test analyses were conducted between models for overall testing accuracy and classification accuracy by tumor type. Boxes with an asterisk indicate p-values < 0.05, which were considered statistically significant. SE: Squeeze-and-Excitation, CBAM: Convolutional Block Attention Module, SA: Self-Attention, AGNet: Attention Gated Network, AUC: area under the receiver operating characteristic curve

Results	SE versus CBAM	SE versus AGNet	SE versus SA	SE versus Base	CBAM versus AGNet	CBAM versus SA	CBAM versus Base	AGNet versus SA	AGNet versus Base	SA versus Base
Test Statistic	3.055	2.775	-0.425	-0.313	-0.313	0.953	-3.305	0.501	0.612	0.112
P-Value	0.002*	0.006*	1.329	1.246	1.245	0.341	1.999	0.616	0.541	0.911
Glioma Test Statistic	1.908	1.667	1.927	1.889	-0.257	0	0	0.263	0.252	0
P-Value	0.056	0.096	0.054	0.059	1.203	1	1	0.793	0.801	1
Meningioma Test Statistic	2.079	1.643	2.823	2.485	-0.494	0.837	0.444	1.333	0.946	-0.407
P-Value	0.038*	0.1	0.005*	0.013*	1.379	0.403	0.657	0.182	0.344	1.316
No Tumor Test Statistic	0	0	0.657	-0.729	0	0.72	-0.76	0.674	-0.737	-1.43
P-Value	1	1	0.511	1.534	1	0.471	1.553	0.501	1.539	1.847
Pituitary Test Statistic	2.038	2.039	2.038	2.423	0	0	0.524	0	0.537	0.53
P-Value	0.042*	0.041*	0.042*	0.015*	1	1	0.6	1	0.591	0.596

## Discussion

The findings of this study demonstrate the significant impact of attention mechanisms in enhancing the performance of the ResNet50V2-based MRI tumor classification model. Among the evaluated frameworks, the SE module emerged as the most effective, achieving the highest testing accuracy (98.4%) and an AUC of 1.00, outperforming the base ResNet50V2 model and other attention-enhanced architectures. The results suggest that integrating attention mechanisms, particularly channel-wise recalibration through SE, can improve feature representation and classification accuracy. This is particularly beneficial in medical imaging tasks, where subtle differences in MRI tumor features can significantly impact diagnosis. The statistical analysis further supports the efficacy of SE, with significant improvements over CBAM (Z = 3.055, p = 0.002) and AGNet (Z = 2.775, p = 0.006) in overall testing accuracy. In tumor-specific classification, SE achieved the highest accuracy across glioma (98%), meningioma (98%), no tumor (97%), and pituitary tumors (100%). The baseline ResNet50V2 model performed notably lower, with 92% accuracy for glioma, 89% for meningioma, 99% for no tumor, and 93% for pituitary tumors. CBAM and AGNet performed similarly, achieving 95% accuracy for pituitary tumors, whereas SA had the lowest accuracy among the attention-based models for meningioma (87%) and no tumor classification (94%). Notably, the baseline model outperformed SA in meningioma and no tumor classification. The implementation of SE mechanisms for improved tumor imaging has also been demonstrated in previous literature. Previous studies have demonstrated high classification accuracy when applying SE attention mechanisms to MRI tumor imaging [10,11]. For example, a SE-enhanced Residual Network 101 (ResNet-101) model by Ghosal et al. in 2019 achieved an overall accuracy of 93.83% in classifying gliomas, meningiomas, and pituitary tumors [10]. Another study by Dash and Sisodia in 2024 demonstrated even higher testing classification accuracy of 98.56% across the same types of tumors using a different type of CNN called Mobile Network Version 1 (MobileNetV1) [11].

While CBAM and AGNet also demonstrated improvements over the baseline model, their performance gains were relatively modest, with the SA model performing worse than the base model, although the differences among all these scores were not statistically significant as shown in Table 2. CBAM, which integrates both channel and spatial attention, achieved a testing accuracy of 93.5%, indicating that spatial attention modulation may not be as important compared to channel-wise feature recalibration in MRI tumor classification. However, a previous model by Shyamala and Mahaboobbasha demonstrated a similar CBAM-integrated ResNet50V2 model that achieved a classification accuracy of 99.33% in identifying low-grade gliomas in an MRI dataset of 110 patients [12]. The likely cause for the discrepancy in accuracies may be because this study implemented a "skull-stripping" preprocessing technique, which removes non-brain tissue (e.g., skull and scalp) before model training [12]. This may enhance performance by reducing background noise and exclusively focusing training on brain tissue and gliomas. Statistically significant differences were observed in overall accuracy between the SE and CBAM models (Z = 3.055, p = 0.002) and between the SE and AGNet models (Z = 2.775, p = 0.006). Additionally, for tumor-specific classification, significant improvements were noted for meningioma classification accuracy, where the SE model outperformed CBAM (Z = 2.079, p = 0.038), SA (Z = 2.823, p = 0.005), and the baseline ResNet50V2 (Z = 2.485, p = 0.013). Similarly, for pituitary tumor classification, the SE model showed statistically significant gains over CBAM (Z = 2.038, p = 0.042), AGNet (Z = 2.039, p = 0.041), SA (Z = 2.038, p = 0.042), and the baseline ResNet50V2 (Z = 2.423, p = 0.015), reinforcing its effectiveness across tumor categories. AGNet, which employs cross-spatial attention and multi-scale feature learning, performed slightly better (94.2%) but did not reach the level of SE. Previous literature on AGNet-enhanced residual network models on MRI brain tumor imaging is relatively sparse, lacking sufficient data to compare this study's current attention-modulated ResNet50V2 variant or instruction on how to improve performance.

Lastly, SA, originally designed for natural language processing, had the lowest accuracy (91.6%), suggesting that its effectiveness in capturing long-range dependencies may not be as crucial for this classification task. Unlike natural language data, which is inherently sequential and benefits from attention mechanisms that track dependencies across distant words, medical imaging relies heavily on spatial and channel-wise feature extraction. MRI tumor classification requires capturing both local texture patterns and global structural variations across multiple channels, making spatial-channel attention mechanisms such as SE and CBAM more effective. Another explanation could be that this model lacks integration within a transformer module, which allows for explicit spatial encoding, leading to suboptimal feature extraction compared to SE and CBAM. Without mechanisms that emphasize spatial hierarchies and multi-channel dependencies, SA may struggle to differentiate tumor characteristics effectively. Supporting this notion is evidence of SA units within transformer mechanisms in a wide range of CNN architectures achieving between 98% and 99% classification accuracy across meningiomas, gliomas, and pituitary tumors [13].

Overall, the SE model exhibited statistically significant improvements in classifying meningiomas and pituitary tumors compared to other models, underlining how an SE framework may enhance the model's ability to distinguish between similar tumor classes, reducing misclassification rates. The lack of significant differences in no tumor classification across models suggests that attention mechanisms primarily enhance feature differentiation for complex tumor patterns. The SE model's superior performance can likely be attributed to its ability to adaptively weigh each channel's contribution, ensuring that critical tumor characteristics receive greater emphasis. Compared to CBAM and AGNet, which incorporate spatial attention, SE's exclusive focus on channel recalibration may explain its advantage. Spatial attention mechanisms prioritize regional dependencies, which, while useful in object detection and natural scene analysis, may not be as crucial in MRI classification, where tumors can appear in various locations but exhibit consistent textural and intensity-based differences across channels. These findings demonstrate that an SE framework may be an ideal attention strategy for MRI-based brain tumor classification.

Despite these promising results, certain limitations should be acknowledged. First, the dataset, while diverse, was sourced from a single publicly available database without access to details regarding sampling methodology, which may limit generalizability. The lack of information on acquisition settings, scanner variability, and potential class imbalances raises concerns about dataset bias, which could impact the model's ability to generalize to underrepresented tumor subtypes. Like all datasets, there is a risk of representation bias, which can arise from intentional or unintentional discrimination in data acquisition and preparation, leading to potential oversights in minority case inclusion and skewing predictive ability for underrepresented demographics [14]. Another limitation lurking in all CNNs is overfitting, a phenomenon in which a model learns nonrepresentative features or noise within the dataset, which possibly hinders the ability to classify images on external datasets outside of what the model was trained on [15]. While training callbacks and dropout regularization were implemented to mitigate this risk, the model was not evaluated on an independent external dataset, leaving the possibility of domain shift bias, where variations in MRI acquisition parameters and preprocessing techniques across institutions could affect classification accuracy. Expanding the dataset to include images from multiple institutions with different imaging protocols would help improve robustness. As this model was not tested on an external dataset, future testing may reveal domain shift bias, in which physical image parameters from image capturing and processing methodologies could skew the model's ability to discriminate among tumor classes, building justification for an enrichment of the dataset using images from different institutions and a variety of patients [16].

To address these intrinsic limitations, future research should validate these findings using larger, multi-institutional datasets to ensure robustness across different imaging protocols and populations. Additionally, advanced domain adaptation techniques, such as adversarial training or contrastive learning, could help mitigate dataset bias and improve generalizability. Implementing self-supervised learning strategies could reduce reliance on labeled data while maintaining classification performance. Furthermore, investigation into artificial dataset image generation using visual autoencoders or generative adversarial networks should be taken into consideration to easily increase dataset size and variation without strenuous effort in data collection, processing, and labeling. Lastly, integrating transformer-based architectures, which have demonstrated strong feature extraction capabilities in medical imaging tasks, could serve as an alternative approach to channel-wise attention mechanisms such as SE, potentially enhancing tumor classification performance. These methodological refinements would not only improve model generalizability but also pave the way for more reliable clinical applications of deep learning in MRI-based tumor classification.

## Conclusions

This study highlights the significant impact of attention mechanisms on enhancing MRI tumor classification performance using a ResNet50V2-based model. The results demonstrate that the SE module provides the most substantial improvement, achieving the highest overall accuracy and AUC scores. The findings emphasize the importance of channel-wise feature recalibration in medical imaging and suggest that SE-enhanced models can serve as powerful tools for automated tumor diagnosis. Future research should explore the integration of hybrid attention mechanisms to further refine classification performance. Additionally, validating these results across larger and more diverse datasets will be crucial to ensuring model generalizability. Given the promising outcomes observed in this study, attention-enhanced deep learning models hold significant potential to improve the accuracy and efficiency of medical image-based models used in research and development.

## Appendices

The dataset used in this study, available on Kaggle, comprises 3,096 annotated MRI scans categorized into four classes: meningioma (n = 913), glioma (n = 901), pituitary tumors (n = 844), and no tumor (n = 438). This dataset provides preprocessed 256 × 256-pixel images with greyscale normalization and image zoom, facilitating deep learning-based tumor classification research. The link to the dataset is as follows: https://www.kaggle.com/datasets/thomasdubail/brain-tumors-256x256/code.
